# Secondary amenorrhea in a woman with spinocerebellar degeneration treated with thyrotropin-releasing hormone: a case report and in vitro analysis

**DOI:** 10.1186/1752-1947-5-567

**Published:** 2011-12-09

**Authors:** Haruhiko Kanasaki, Aki Oride, Tselmeg Mijiddorj, Indri Purwana, Kohji Miyazaki

**Affiliations:** 1 Department of Obstetrics and Gynecology, Shimane University School of Medicine, Izumo 693-8501, Japan

## Abstract

**Introduction:**

While thyrotropin-releasing hormone is known to be a prolactin-release stimulating factor, thyrotropin-releasing hormone-tartrate and its derivative, taltirelin hydrate, are used for the treatment of spinocerebellar degeneration, a degenerative disease characterized mainly by motor ataxia. We report the case of a patient being treated with a thyrotropin-releasing hormone preparation for spinocerebellar degeneration who developed amenorrhea after a dose increase. Her hormonal background was analyzed and the effect of prolonged exposure to thyrotropin-releasing hormone on pituitary prolactin-producing cells was examined *in vitro*.

**Case presentation:**

Our patient was a 36-year-old Japanese woman who experienced worsening of gait disturbance at around 23 years of age, and was subsequently diagnosed as having spinocerebellar degeneration. She had been treated with thyrotropin-releasing hormone-tartrate for four years. Taltirelin hydrate was added to the treatment seven months prior to her presentation, followed by an improvement in gait disturbance. Around the same period, she started lactating and subsequently developed amenorrhea three months later. Taltirelin hydrate was discontinued and she was referred to our hospital. She was found to have normal sex hormone levels. A thyrotropin-releasing hormone provocation test showed a normal response of thyroid-stimulating hormone level and an over-response of prolactin at 30 minutes (142.7 ng/mL). Resumption of menstruation was noted three months after dose reduction of thyrotropin-releasing hormone. In our *in vitro *study, following long-term exposure to thyrotropin-releasing hormone, cells from the rat pituitary prolactin-producing cell line GH3 exhibited an increased basal prolactin promoter activity but showed a marked decrease in responsiveness to thyrotropin-releasing hormone.

**Conclusions:**

Physicians should be aware of hyperprolactinemia-associated side effects in patients receiving thyrotropin-releasing hormone treatment. Long-term treatment with a thyrotropin-releasing hormone preparation might cause a large amount of prolactin to accumulate in prolactin-producing cells and be released in response to exogenous thyrotropin-releasing hormone stimulation.

## Introduction

Thyrotropin-releasing hormone (TRH) is a hypothalamic hormone discovered as a peptide that promotes the release of thyroid-stimulating hormone (TSH) from the anterior pituitary gland [1]. TRH is also known to be a prolactin (PRL)-releasing factor [2] and the TRH provocation test is widely performed to evaluate the ability to secrete PRL. TRH-tartrate (TRH-T) is used as a diagnostic agent for hyperprolactinemia in gynecological practice. TRH-T has also been shown to be effective in improving gait disturbance and trunk sway caused by spinocerebellar degeneration (SCD), an intractable neurological disorder, and has been widely used in clinical practice [3]. Taltirelin hydrate was subsequently developed as an orally administered TRH derivative and has been used as a safe central nervous system (CNS)-selective agent for the treatment of SCD [4,5]. Both TRH-T and taltirelin hydrate are considered to activate the signal transduction system of the CNS. At the same time, both agents mimic TRH and thus may affect endocrine function in women. We describe here the case of a patient with SCD who developed secondary amenorrhea suspected to have been induced by the use of TRH-T and its derivative taltirelin hydrate.

## Case presentation

Our patient was a 36-year-old Japanese woman who had been pregnant three times and had given birth to three children. She had experienced mild gait disturbance since she was around two years of age but the cause had remained uncertain. She noted worsening of the gait disturbance around age 23 and was subsequently diagnosed as having SCD at age 31 when she gave birth to her second child through vaginal delivery. Treatment was started with 2 mg of TRH-T (Hirtonin, Takeda Pharmaceutical Co., Osaka, Japan) given intravenously once or twice a month. Taltirelin hydrate (10 mg/day) (Ceredist, Tanabe-Mitsubishi Pharma Corp., Osaka, Japan) was added to the treatment one year prior to presentation, followed by a significant improvement in gait disturbance. Around the same time, she started lactating and experiencing irregular menstruation. Three months after the addition of taltirelin hydrate, she developed amenorrhea and presented at this time to our obstetrics and gynecological department. Her hematological test results at presentation are summarized in Table 1. On examination, no abnormal findings were observed in either sex hormone levels (luteinizing hormone (LH) 9.6 mIU/mL, follicle-stimulating hormone (FSH) 5.7 mIU/mL, estradiol (E2) 69 pg/mL and PRL 11.2 ng/mL) or thyroid function (fT3 2.2 pg/mL, fT4 0.9 nf/dL, TSH 1.26 μU/mL). Complete blood count and biochemical tests were also normal. Pelvic examination and endovaginal ultrasonography revealed no abnormalities in the uterus or ovaries, such as polycystic ovary.

**Table 1:** Laboratory findings

Parameter	Value
White blood cell count	3220 cells/μL
Red blood cell count	4.04 × 106 cells/μL
Hemoglobin	11.9 g/dL
Hematocrit	36.7%
Platelets	171 × 10^3 ^cells/μL
Total protein	6.6 g/dL
Albumin	4.3 g/dL
Total bilirubin	0.7 mg/dL
Aspartate aminotransferase	15 IU/L
Alanine aminotransferase	13 IU/L
Blood urea nitrogen	11.2 mg/dL
Creatine	0.58 mg/dL
Na	143 mEq/L
K	3.9 mEq/mL
Cl	105 mEq/mL
Free T3	2.2 pg/mL
Free T4	0.9 ng/dL
Thyroid-stimulating hormone	1.26 μU/mL
Leutenizing hormone	9.6 mIU/mL
Follicle-stimulating hormone	5.7 mIU/mL
Estradiol	69 mIU/mL
Prolactin	11.2 ng/mL

**Figure 1:**
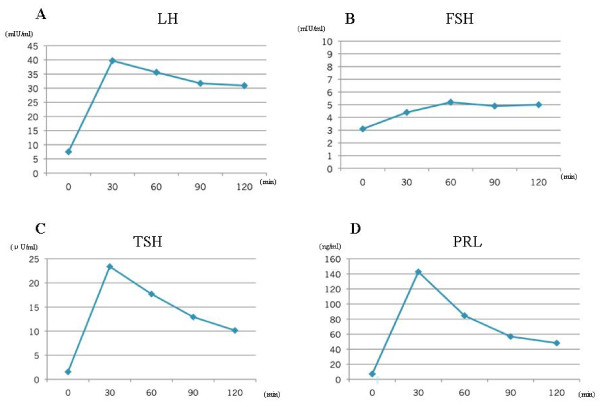
**Luteinizing hormone releasing hormone (LH-RH) and thyrotropin-releasing hormone (TRH) provocation test results from our patient**. LH **(A) **and follicle-stimulating hormone (FSH) **(B) **release by LH-RH provocation test. Thyroid-stimulating hormone (TSH) **(C) **and prolactin (PRL) **(D) **release by TRH provocation test.

Administration of E2 and progesterone induced withdrawal bleeding. The hormone provocation test was performed five days after the start of this bleeding and the results are summarized in Figure 1. Her LH level was 7.5 mIU/mL at baseline and increased to 39.7, 35.9, 31.7, and 30.9 mIU/mL at 30, 60, 120, and 180 minutes, respectively, after provocation with 100 μg LH-releasing hormone (RH) (Figure 1A). Her basal FSH level was 3.1 mIU/mL and the levels at the corresponding time points after LH-RH provocation were 4.4, 5.2, 4.9, and 5.0 mIU/mL (Figure 1B). Her basal LH level was higher than her basal FSH level, and her LH level, but not her FSH level, showed a mild over-response to the provocation with LH-RH. In the provocation test with 500 μg TRH, her TSH level showed a normal response with levels of 1.55 μU/mL at baseline and 23.4, 17.69, 12.93, and 10.15 μU/mL at 30, 60, 90, and 120 minutes, respectively, after provocation (Figure 1C), whereas her PRL level exhibited an over-response with levels of 7.1 ng/mL at baseline and 142.7, 84.6, 56.8, and 48.2 ng/mL at 30, 60, 90, and 120 minutes, respectively, after provocation (Figure 1D). The results led to a diagnosis of latent hyperprolactinemia. An MRI scan of the head was performed but revealed no abnormal findings in her pituitary gland. Given that withdrawal bleeding was induced by the administration of an E2/progesterone preparation, it was explained to our patient that regular induction of withdrawal bleeding was considered necessary. Taltirelin hydrate (Ceredist) was discontinued at the discretion of a physician, followed by the return of menstruation three months later. This also resulted in cessation of lactation.

**Figure 2:**
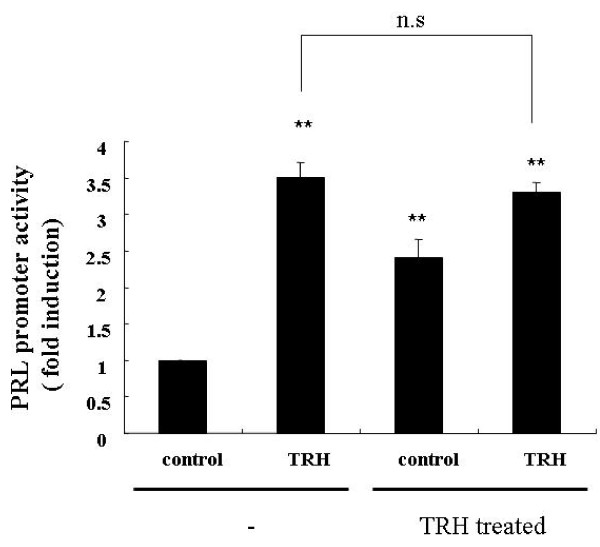
**Response to thyrotropin-releasing hormone (TRH) on prolactin promoter activity in TRH-treated cells**. GH3 cells were treated or non-treated with 100 nM TRH for 48 hours in 1% fetal bovine serum (FBS)-containing Dulbecco's modified Eagle medium (DMEM). Then, the culture medium was washed with and changed to serum-free DMEM. Next, the cells were stimulated with 100 nM TRH for six hours. Luciferase activity was measured and relative induction is shown (relative to TRH-untreated control set as 1.0). **P < 0.01 versus controls in TRH non-treated cells. The difference between TRH stimulation in non-treated cells and treated cells was not statistically significant.

Our patient showed a normal basal PRL level following long-term administration of the TRH agonist, but exhibited over-response of PRL in the provocation test. We subsequently investigated the effect of long-term administration of TRH on PRL-producing cells using GH3 cells, a rat-derived PRL-producing cell line. GH3 cells were pre-cultured in the presence or absence of 100 nM TRH for 48 hours. After the culture medium was exchanged (TRH was removed), the cells were continuously stimulated with 100 nM TRH for six hours and measured for PRL promoter activity. The cells that were not exposed to the 48-hour pre-treatment with TRH exhibited a 3.51 ± 0.20-fold increase in PRL promoter activity in response to TRH stimulation, compared with controls. For the cells pre-treated with TRH for 48 hours (TRH-treated cells), basal promoter activity increased by 2.41 ± 0.25-fold and the activity increased by 3.31 ± 0.42-fold following stimulation with TRH (rates of increase were relative to the TRH-untreated control set as 1.0). The PRL promoter activity of the TRH-treated cells increased by 1.37-fold following TRH stimulation (3.31/2.41 = 1.37) (Figure 2).

## Discussion

Gynecologists generally recognize TRH as the agent used in the TRH provocation test, but are not necessarily aware that this agent is also routinely used for the treatment of SCD, a neurological disorder. Our patient had been diagnosed as having SCD and treated with TRH-tartrate for a long period until she subsequently started receiving another TRH agonist, taltirelin hydrate, and developed amenorrhea. Although the basal secretion level of PRL was within the normal range, an over-response in PRL secretion was noted in the TRH provocation test, suggesting latent hyperprolactinemia. TRH, a hypothalamic hormone, was originally discovered as a central factor that stimulates the anterior pituitary gland to secrete TRH and thereby regulates thyroid hormone secretion [1]. The hormone was later found to promote PRL secretion when applied to rat pituitary cells [6] or when intravenously infused to humans [7]. Since then, the hormone has also been recognized as a substance for promoting the secretion of PRL. Therefore, at the initial presentation of our patient to our hospital, we speculated that the excessive dosing of TRH might have induced hyperprolactinemia and subsequent amenorrhea. However, a sex hormone test at that time showed no evidence of hyperprolactinemia, with a PRL level of 11.2 ng/mL, which was within the normal range. Although a high basal LH level was noted (LH 9.6 mIU/mL, FSH 5.7 mIU/mL), transvaginal ultrasonography did not show polycystic ovary, which did not meet the new criteria for diagnosing polycystic ovarian syndrome as proposed by the Reproduction and Endocrine Committee of the Japan Society of Obstetrics and Gynecology (2007). Given that a hormone provocation test showed a marked increase in the PRL level from 7.1 ng/mL before provocation to 142.7 ng/mL 30 minutes after provocation with TRH, our patient was considered to have latent hyperprolactinemia with abnormal lactation and menstruation. What are considered normal values for serum PRL differs according to the assay system used. The chemiluminescence immunoassay (CLIA) we currently use defines normal serum PRL levels as 3.2 to 26.2 ng/mL. Although there are no standard criteria for the diagnosis of latent hyperprolactinemia using recent assay systems, we diagnosed latent hyperprolactinemia based on the observation that serum PRL increased to more than 140 ng/mL after the TRH provocation test, serum PRL was increased more than 20-fold by TRH, and that our patient had symptoms associated with hyperprolactinemia.

We then examined whether this *in vivo *condition can be reproduced *in vitro *using prolactin-producing cells. GH3 cells are somatolactotrophs derived from rat pituitary tumor and produce/secrete PRL and growth hormone (GH). These cells are widely used as model cells for the elucidation of the regulation mechanism of PRL production, as they share many characteristics with normal lactotrophs and somatolactotrophs in addition to secreting PRL and GH in response to stimulation with TRH [8,9]. To examine the effect of long-term stimulation with TRH on GH3 cells, we cultured GH3 cells in the presence of TRH for 48 hours and evaluated their responsiveness to TRH stimulation by measuring PRL promoter activity. The GH3 cells continuously stimulated with TRH for 48 hours showed a 2.4-fold increase in basal PRL promoter activity compared with TRH-untreated control cells. This indicated that the PRL promoter was activated by persistent stimulation with TRH, leading to the activation of intracellular PRL synthesis. Intracellularly produced PRL proteins are released as secretary granules in response to TRH while maintaining a certain level of basal secretion. Given the results of the *in vitro *experiment, it was expected that long-term treatment with TRH would cause hyperprolactinemia. However, our patient showed no increase in the basal PRL level even after continuous treatment with TRH. The cells pre-treated with TRH for 48 hours only exhibited a 1.37-fold increase in PRL secretion compared with the controls in response to subsequent TRH stimulation. *In vivo *(that is, in our patient), the basal PRL level was 7.1 ng/mL, which was within the normal range, but increased by about 20-fold to 142.7 ng/mL 30 minutes after TRH stimulation. Thus, different responses to stimulation following continuous administration of TRH were observed *in vitro *and *in vivo*.

Although it is not possible to make a direct comparison of the *in vitro *and *in vivo *responses (because PRL secretion was measured *in vivo *while PRL promoter activity was measured *in vitro*), there was at least a difference in the response to TRH after long-term exposure to it.

*In vivo*, the secretion and synthesis of PRL have been shown to be down-regulated mainly by dopamine D2 receptor agonists [10] and this mechanism is utilized in the treatment of pituitary tumor and other therapeutic applications. PRL has also been known to be up-regulated by various hypothalamic hormones other than TRH or anterior pituitary hormones [11]. The *in vitro *experiment in this study only evaluated the effect of long-term TRH treatment on homogeneous PRL-producing cells and did not consider its effects on other hypothalamic hormones, such as dopamine, or cells secreting hormones other than PRL. Moreover, our patient had been treated with TRH-T (Hirtonin) alone for four years before combination therapy with Hirtonin and taltirelin hydrate (Ceredist) was started. In the *in vitro *experiment, however, a 48-hour treatment of cells with TRH was used to model the long-term administration of TRH in our patient. This might have contributed to the differences observed *in vitro *and *in vivo*. At the same time, *in vivo *PRL transcription activity was increased by approximately 2.5-fold following the 48-hour treatment with TRH. TRH stimulation activates various signal transmitters. In particular, the activation of extracellular signal-regulated kinase (ERK) mediated by protein kinase C (PKC) plays an important role in the expression of the PRL gene. Therefore, PRL gene expression is suppressed by inhibitors of these kinases. However, studies examining TRH-mediated PRL secretion have shown that PRL secretion is not suppressed by PKC/ERK inhibitors, but is instead strongly suppressed by calcium-dependent kinase inhibitors [8]. Our patient showed no increase in basal PRL level but showed an over-response to TRH. This can possibly be attributed to the long-term treatment with a TRH preparation that might have caused the accumulation of a large amount of PRL in PRL-producing cells and subsequent massive release of the hormone in response to exogenous TRH stimulation.

## Conclusions

We report the case of a patient who was treated with a TRH preparation for SCD who developed amenorrhea following a dose increase. Our patient had a normal PRL level but showed a response pattern suggestive of latent hyperprolactinemia following stimulation with TRH. Physicians should be cognizant of hyperprolactinemia-associated side effects in patients receiving TRH treatment. The experiment using PRL-producing cells demonstrated that long-term exposure to TRH resulted in increased basal activity of PRL synthesis and decreased responsiveness to TRH. Thus, different responses to TRH were observed *in vivo *and *in vitro *following the long-term administration of TRH.

## Consent

Written informed consent was obtained from the patient for publication of this manuscript and any accompanying images. A copy of the written consent is available for review by the Editor-in-Chief of this journal.

## Competing interests

The authors declare that they have no competing interests.

## Authors' contributions

HK, AO, and KM analyzed and interpreted the data from our patient regarding clinical course and outcome. TM and IP performed the *in vitro *analysis. HK was a major contributor to writing the manuscript. All authors read and approved the final manuscript.
